# Oral microbiota in the oral-genitourinary axis: identifying periodontitis as a potential risk of genitourinary cancers

**DOI:** 10.1186/s40779-021-00344-1

**Published:** 2021-09-29

**Authors:** Shuai Yuan, Cheng Fang, Wei-Dong Leng, Lan Wu, Bing-Hui Li, Xing-Huan Wang, Hailiang Hu, Xian-Tao Zeng

**Affiliations:** 1 Center for Evidence-Based and Translational Medicine, Zhongnan Hospital of Wuhan University, Wuhan, 430071 Hubei China; 2 Department of Stomatology, Taihe Hospital, Hubei University of Medicine, Shiyan, 442000 Hubei China; 3 Department of Stomatology, Zhongnan Hospital of Wuhan University, Wuhan, 430071 Hubei China; 4 Department of Urology, Zhongnan Hospital of Wuhan University, Wuhan, 430071 Hubei China; 5 Department of Pathology, Duke University School of Medicine, Durham, NC 27710 USA; 6 School of Medicine, Southern University of Science and Technology, Shenzhen, 518055 China

**Keywords:** Oral microbiota, Oral-genitourinary axis, Periodontitis, Urogenital neoplasms, Prostatic neoplasms, Kidney neoplasms, Urinary bladder neoplasms

## Abstract

Periodontitis has been proposed as a novel risk factor of genitourinary cancers: although periodontitis and genitourinary cancers are two totally distinct types of disorders, epidemiological and clinical studies, have established associations between them. Dysbiosis of oral microbiota has already been established as a major factor contributing to periodontitis. Recent emerging epidemiological evidence and the detection of oral microbiota in genitourinary organs indicate the presence of an oral-genitourinary axis and oral microbiota may be involved in the pathogenesis of genitourinary cancers. Therefore, oral microbiota provides the bridge between periodontitis and genitourinary cancers. We have carried out this narrative review which summarizes epidemiological studies exploring the association between periodontitis and genitourinary cancers. We have also highlighted the current evidence demonstrating the capacity of oral microbiota to regulate almost all hallmarks of cancer, and proposed the potential mechanisms of oral microbiota in the development of genitourinary cancers.

## Background

Periodontal disease is an inflammatory and multifactorial chronic immunological disease consisting of two main subtypes, gingivitis and periodontitis [1–4]. Gingivitis is a reversible inflammation of the gingiva (gums, soft tissue surrounding the teeth) alone while periodontitis is characterized by permanent loss of periodontal tissue support [5]. In particularly susceptible individuals with compromised immune systems, gingivitis progresses to periodontitis, a chronic, irreversible breakdown of soft and hard tissues surrounding the teeth [1–4]. Periodontitis has been raised as a public health problem: an estimated 42.2% of dentate adults aged 30 years or older in the United States have periodontitis, with 7.8% having severe periodontitis [6]. The prevalence of severe periodontitis varies in different countries and affects 11% of the global population [7]. Numerous studies have established associations between periodontitis and many systemic diseases, including cancer [8–12]. However, the precise mechanisms underlying these relationships remain unclear. As dysbiosis of oral microbiota has been involved in the pathogenesis of both periodontitis and systemic diseases, the causative link between periodontitis and systemic diseases, including cancer, through oral microbiota is being extensively explored [13, 14].

The microbiota present in tissues or mucosal sites have been shown to be involved in carcinogenesis and modulate the responses to cancer therapy [15, 16]. The oral cavity is connected to the respiratory tract and acts as the entry portal for the gastrointestinal tract. Oral microbiota residing in the oral cavity have the opportunity to enter the respiratory and gastrointestinal tract. Thus, it is not surprising that dysbiosis of oral microbiota in periodontal disease, especially in periodontitis, can contribute to cancers of the gastrointestinal and respiratory tract, such as head and neck squamous cell carcinoma, lung cancer, pancreatic cancer, gastric cancer, and colorectal cancer [17–22]. In addition to the above tumors, emerging epidemiological evidence suggests that periodontitis is also associated with the risk of genitourinary cancers, such as prostate and bladder cancer [23, 24]. The recent discovery of pathogenic bacterial infections in genitourinary cancers highlights the tumor-promoting effect of the microbiome in prostate, bladder, and kidney cancers [25]. Ten hallmarks of cancer have been proposed by Hanahan and Weinberg to better our understanding of the complex multistep changes occurring during cancer development [26, 27]. These cancer hallmarks can basically be considered as the consequence of the tumor and tumor microenvironment interactions and the microbiota has been shown to be an important player in the homeostasis of the tumor-microenvironment, thus modulating almost all cancer hallmarks [28]. Given that oral microbiota can be detected in the genitourinary system [29], it is highly likely that they may promote the progression of genitourinary cancers and therefore provide the bridge between periodontitis and genitourinary cancers.

In this narrative review, we will summarize the epidemiological studies exploring the association between periodontitis and genitourinary cancers and highlight the current evidence for oral microbiota regulating the hallmarks of cancer, which may suggest the potential molecular mechanisms of oral microbiota in genitourinary cancers.

## Methods

The aims of this narrative review are to: (1) review epidemiologic studies exploring the association between periodontitis and genitourinary cancers; (2) review the potential mechanisms of oral microbiota in genitourinary cancers. According to the National Cancer Institute (NCI) [30], genitourinary cancers comprise cancers found in the urinary system and the male reproductive system, including bladder cancer, kidney (renal cell) cancer, penile cancer, prostate cancer, transitional cell cancer of the renal pelvis and ureter, testicular cancer, urethral cancer, Wilms tumor, and other childhood kidney tumors.

To review epidemiologic studies exploring the association between periodontitis and genitourinary cancers we used the following inclusion criteria: (1) Participants: bladder cancer, kidney (renal cell) cancer, penile cancer, prostate cancer, transitional cell cancer of the renal pelvis and ureter, testicular cancer, urethral cancer, Wilms tumor, and other childhood kidney tumors; (2) Exposure: periodontitis, periodontal disease; (3) Control: without periodontal disease; (4) Outcome: initiation (incidence, formation of new tumors) and progression (further development of already existing tumors) of genitourinary cancers; (5) Study design: cohort study or case–control study. Cross-sectional studies were excluded. In the literature we searched, human papillomavirus (HPV) infections were sexually transmitted and were likely to be transient members of the oral microbiota. Although HPV could be detected in oral and genitourinary cancers, HPV was not in the scope of our review. Apart from HPV, we did not find any evidence that oral microbiota linked periodontitis to penile cancer, transitional cell cancer of the renal pelvis and ureter, testicular cancer, urethral cancer, Wilms tumor or childhood kidney tumors. Therefore, we only focused on prostate cancer, bladder cancer and kidney cancer in this review.

To review the potential mechanisms of oral microbiota in genitourinary cancers. We conducted a literature search using PubMed, Embase and Web of Science to identify relevant research studies and review articles published in English up to December 2020 using the following keywords: periodontal disease, periodontitis, periodontal bacteria, periodontal pathogens, oral pathogens, oral microbiota, oral microbiome, oral microflora, oral bacteria, subgingival microflora, cancer, genitourinary cancers, prostate cancer, bladder cancer, urothelial carcinoma, kidney cancer, and renal cell carcinoma. We focused on in vitro and in vivo studies to propose the potential mechanisms of oral microbiota in the development of genitourinary cancers.

## Epidemiological associations between periodontitis and genitourinary cancers

The link between periodontitis and increased risk of genitourinary cancers has been established by epidemiological and clinical studies, suggesting that periodontitis is a novel risk factor for genitourinary cancers (Table 1). Many epidemiological studies have not clearly distinguished between gingivitis and periodontitis, which were collectively referred to as periodontal diseases. However, it is not likely that simple marginal gingivitis caused by supragingival plaque has much systemic effect, except for some inflammatory biomarkers that may be found in both the gingiva and the bloodstream. Periodontitis, by contrast, is accompanied by the pathogenic anaerobic bacteria that are usually reported to be associated with remote tumors and tumorigenesis. It should also be noted that there was not any global definition of periodontitis until the Centers for Disease Control and Prevention (CDC) and the American Academy of Periodontology (AAP) published such classifications for population-based surveillance in 2007 (no/mild, moderate, and severe periodontitis) [31] and in 2012 (no, mild, moderate, and severe periodontitis) [32]. Such a lack of case definitions leads to the heterogeneity of the definition of periodontitis in population-based studies [31–37], which could hamper establishing epidemiological associations between periodontitis and genitourinary cancers.

**Table 1:** Epidemiological studies exploring the associations between periodontitis and genitourinary cancers

Author (year)	PD criteria	Cases N (M/F)	Controls N (M/F)	Results
*Prostate cancer*				
Michaud et al. (2016) [23]	Self-reported periodontitis according to bone loss and teeth number [33, 36]	Periodontitis: 1945 (M: 1945)	Without PD: 17,988 (M: 17,988)	(1) No significant association between periodontitis and PCa risk (*HR* = 1.17, 95% CI 0.94–1.47) (2) No significant association between tooth loss and PCa risk (*HR* = 0.89, 95% CI 0.61–1.30)
Dizdar et al. (2017) [24]	CDC/AAP case definition [31]	M/S periodontitis: 129 (M: 129)	TNCR 2013 data	M/S periodontitis had higher risk of PCa (*SIR* = 3.75, 95% CI 0.95–10.21)
Kim et al. (2020) [38]	CDC/AAP case definition [31]	CP: 60,772 (M: 60,772)	Without PD: 60,468 (M: 60,468)	CP significantly increased the risk of PCa (*HR* = 1.24, 95% CI 1.16–1.32)
Güven et al. (2019) [39]	AAP classification [35]	PD: 2151 (M: 2151)	TNCR 2013 data	PD significantly increased the risk of PCa (*SIR* = 1.84, 95% CI 1.34–2.49)
Lee et al. (2017) [40]	CDC/AAP case definition [31]	PD: 531 (M: 531)	Without PD: 403 (M: 403)	PD significantly increased the risk of PCa (*HR* = 1.14, 95% CI 1.01–1.31)
Arora et al. (2010) [41]	Self-reported PD according to tooth mobility [34]	PD: 457 (M: 457)	Without PD: 5515 (M: 5515)	PD significantly increased the risk of PCa (*HR* = 1.47, 95% CI 1.04–2.07)
Hujoel et al. (2003) [42]	Gingival inflammation, periodontal pockets, firmness of tooth	Periodontitis: 1085 (M: 1085) Gingivitis: 1021 (M: 1021)	Without PD: 1242 (M: 1242)	Periodontitis was positively associated with PCa (*OR* = 1.81, 95% CI 0.76–4.34)
Hiraki et al. (2008) [43]	Self-reported tooth loss	Pca: 136 (M: 136)	Without cancer: 5398 (M: 5398)	Tooth loss was inversely associated with PCa (*OR* = 0.49, 95% CI 0.19–1.26)
Michaud et al. (2008) [44]	Self-reported PD according to bone loss and teeth number [33, 36]	PD: 7863 (M: 7863)	Without PD: 40,512 (M: 40,512)	(1) No significant association was observed between PD and advanced PCa risk (*HR* = 0.89, 95% CI 0.71–1.10) (2) Tooth loss was inversely correlated with advanced PCa (*HR* = 0.70, 95% CI 0.50–0.97)
Hwang et al. (2014) [45]	NR	PD with treatment: 19,226 (M: 19,226)	PD with no treatment: 38,452 (M: 38,452)	The risks of PCa were significantly higher in the PD with treatment (*HR* = 2.11, 95% CI 1.63–2.73)
Wen et al. (2014) [46]	NR	Periodontitis: 26,288 (M: 26,288)	Gingivitis: 47,522 (M: 47,522)	The incidence rate of PCa was not elevated in the periodontitis cohort than in the gingivitis cohort (*HR* = 1.00, 95% CI 0.82–1.23)
Michaud et al. (2018) [47]	CDC/AAP case definition [32]	Periodontitis: 2252 (M: 2252)	Without Periodontitis: (M: 535)	No significant association was observed between SP and PCa risk (*HR* = 1.10, 95% CI 0.79–1.54)
*Bladder cancer*				
Michaud et al. (2016) [23]	Self-reported periodontitis according to bone loss and teeth number [33, 36]	Periodontitis: 1945 (M: 1945)	Without PD: 17,988 (M: 17,988)	(1) Periodontitis increased the risk of bladder cancer (*HR* = 1.38, 95% CI 0.93–2.05) (2) Advanced periodontitis significantly increased the risk of bladder cancer (*HR* = 5.06, 95% CI 2.32–11.0)
Arora et al. (2010) [41]	Self-reported PD according to Tooth mobility [34]	PD: 908 (M: 457/F:451)	Without PD: 12,592 (M: 5515/F: 7077)	PD slightly increased the risk of bladder cancer (*HR* = 1.13, 95% CI 0.59–2.20)
Michaud et al. (2008) [44]	Self-reported PD according to bone loss and teeth number [33, 36]	PD: 7863 (M: 7863)	Without PD: 40,512 (M: 40,512)	PD slightly increased the risk of bladder cancer (*HR* = 1.17, 95% CI 0.96–1.43)
Wen et al. (2014) [46]	NR	Periodontitis: 57,591 (M: 26,288/F: 25,503)	Gingivitis: 96,375 (M: 47,522/F: 45,583)	The incidence rate of bladder cancer was not elevated in the periodontitis cohort than in the gingivitis cohort (*HR* = 0.93, 95% CI 0.70–1.22)
Oh et al. (2020) [48]	Self-reported PD according to bone loss and teeth number [33, 36]	PD: 89,170 PYs (M: 89,170 PYs)	Without PD: 354,997 PYs (M: 354,997 PYs)	PD increased the risk of invasive bladder cancer (*HR* = 1.19, 95% CI 0.98–1.46)
Nwizu et al. (2017) [49]	Self-reported PD [37]	PD: 17,103 (F: 17,103)	Without PD: 48,766 (F: 48,766)	PD slightly increased the risk of bladder cancer (*HR* = 1.10, 95% CI 0.81–1.49)
*Kidney cancer*				
Michaud et al. (2016) [23]	Self-reported periodontitis according to bone loss and teeth number [33, 36]	Periodontitis: 1945 (M: 1945)	Without PD: 17,988 (M: 17,988)	No association between periodontitis and the risk of kidney cancer (*HR* = 1.09, 95% CI 0.68–1.75)
Michaud et al. (2008) [44]	Self-reported PD according to bone loss and teeth number [33, 36]	PD: 7863 (M: 7863)	Without PD: 40,512 (M: 40,512)	PD significantly increased risk of kidney cancer (*HR* = 1.49, 95% CI 1.12–1.97)
Wen et al. (2014) [46]	NR	Periodontitis: 57,591 (M: 26,288/F: 25,503)	Gingivitis: 96,375 (M: 47,522/F: 45,583)	The incidence rate of kidney cancer was not elevated in the periodontitis cohort than in the gingivitis cohort (*HR* = 0.89, 95% CI 0.65–1.23)
Nwizu et al. (2017) [49]	Self-reported PD [37]	PD: 17,103 (F: 17,103)	Without PD: 48,766 (F: 48,766)	PD slightly increased the risk of kidney cancer (*HR* = 1.09, 95% CI 0.76–1.56)

*AAP* American Academy of Periodontology, *CDC* centers for disease control, *CP* chronic periodontitis, *CI* confidence interval, *F* female, *HR* Hazard ratio, *M* male, *M/S* moderate to severe, *N* number, *NR* not reported, *OR* Odds ratio, *PCa* prostate cancer, *PD* periodontal disease, *PYs* person-years, *SIR* standardized incidence rates, *SP* severe periodontitis, *TNCR* Turkish National Cancer Registry

### Prostate cancer

Mounting evidence suggests that periodontitis may be a potential risk for prostate cancer. A recent cohort study evaluated the cancer risk in patients with chronic periodontitis and suggested that chronic periodontitis significantly increased the risk of prostate cancer [hazard ratio (*HR*) = 1.24, 95% confidence interval (CI) 1.16–1.32] [38]. Two studies in Turkey also found significant correlations between periodontal disease (especially moderate-severe periodontitis) and prostate cancer [24, 39]. Another retrospective cohort study found that periodontal disease increased the risk of prostate cancer by 14% (*HR* = 1.14, 95% CI 1.01–1.31) after adjustment for sociodemographic factors, comorbidities, smoking status, alcohol intake, and regular exercise [40]. One prospective co-twin study reported an association of periodontal disease with the increased risk of prostate cancer (*HR* = 1.47, 95% CI 1.04–2.07) [41] and another study suggested individuals with periodontitis had elevated risk of prostate cancer [odds ratio (*OR*) = 1.81, 95% CI 0.76–4.34] [42] (Table 1).

However, inverse associations or no associations between periodontitis and risk of prostate cancer have also been identified in several studies. One case–control study found tooth loss was inversely associated with the risk of prostate cancer (*OR* = 0.49, 95% CI 0.19–1.26) [43] and another study also reported a significant inverse association between tooth loss and advanced prostate cancer (*HR* = 0.70, 95% CI 0.51–0.97) [44]. Tooth loss can be caused not only by periodontitis but also by caries, trauma, and other reasons, which may have led to these inconsistent epidemiological findings. To complicate the results of association, one study found a significantly higher risk for prostate cancer following the treatment of periodontal disease, supporting the inverse association between periodontal disease and prostate cancer [45]. Several studies have found no associations between periodontitis and the risk of prostate cancer [23, 46, 47] (Table 1). The discrepancy of the findings may be attributed to many factors, such as the different clinical criteria for the diagnosis of periodontitis and the confounders in the epidemiological studies [23, 39, 40, 44].

### Bladder cancer

Periodontitis may also influence the risk of bladder cancer. A recent prospective study found a high risk of bladder cancer in men with a history of periodontal disease alone (*HR* = 1.19, 95% CI 0.98–1.46), and a higher bladder cancer risk in men with a history of both peptic ulcer and periodontal disease (*HR* = 1.52, 95% CI 1.05–2.20) [48]. Prior to this study, four prospective cohort studies revealed a positive but non-significant association of periodontitis (or periodontal disease) with the risk of bladder cancer [23, 41, 44, 49]. One retrospective cohort study, which is as controversial as risks for prostate cancer, did not find any association between periodontitis and the risk of bladder cancer [46] but advanced periodontitis in people who had never smoked was associated with an elevated risk of bladder cancer (HR = 5.06, 95% CI 2.32–11.0) [23] (Table 1).

### Kidney cancer

At present, there are relatively few studies reporting on the effects of periodontitis on kidney cancer (Table 1). Michaud et al. reported that periodontal disease was positively associated with the risk of kidney cancer in the Health Professionals Follow-up Study [44]. However, after an additional 8 years of follow-up in the same cohort, no associations were observed between periodontitis and the risk of kidney cancer among males who had never smoked [23]. Another two studies also found the incidence rate of kidney cancer was not elevated in the periodontitis (or periodontal disease) cohort [46, 49] (Table 1). Further studies are needed to examine the correlation between periodontitis and kidney cancer.

## Dysbiosis of oral microbiota in periodontitis

**Fig. 1:**
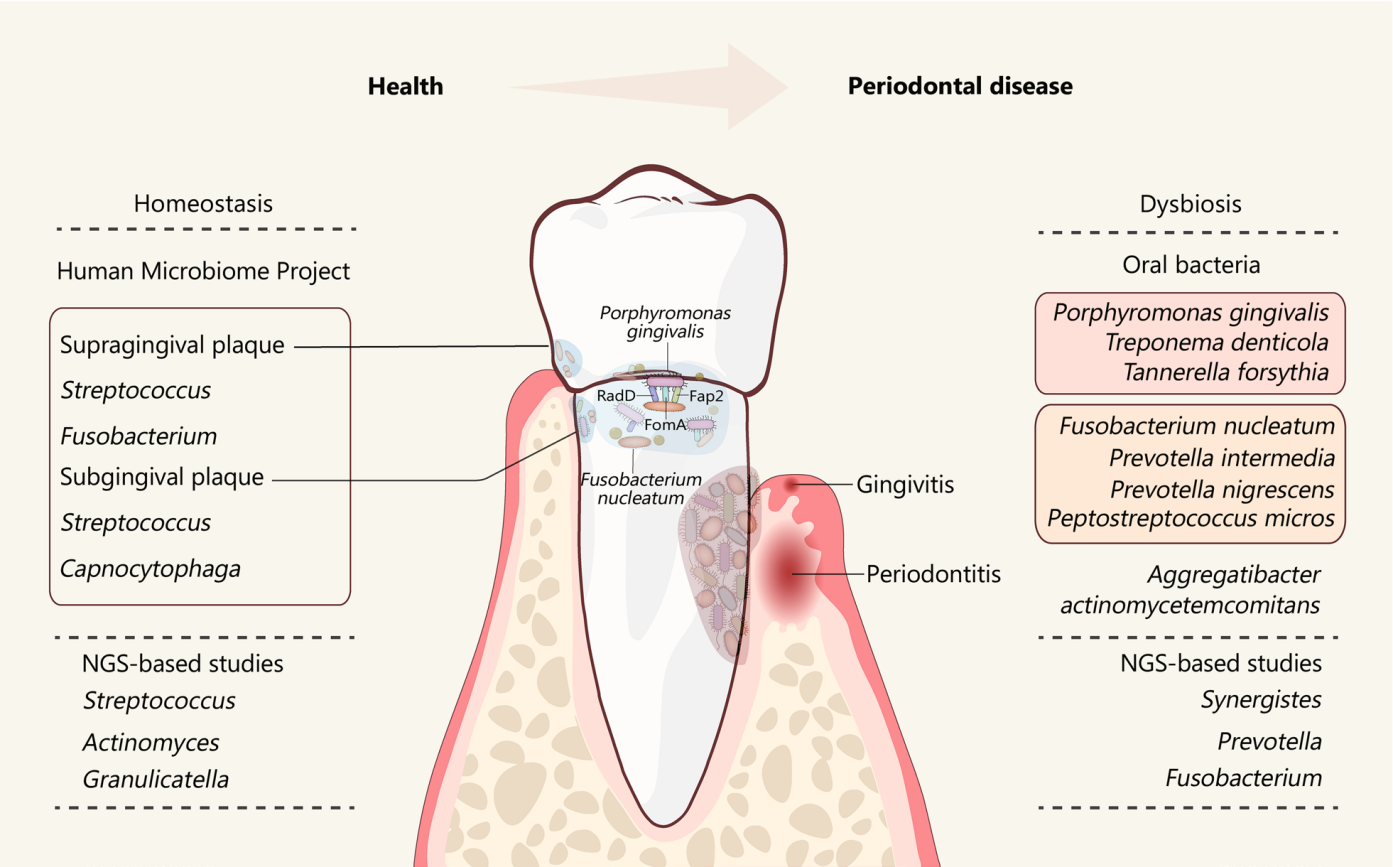
Oral microbiota in periodontal health (left) and disease (right). The predominant bacteria identified by Human Microbiome project or NGS-based studies in periodontal health (left) and periodontal disease (right) are shown in the figure. *F. nucleatum* acts as a mutualist in healthy periodontal tissue. In periodontitis, *F. nucleatum* turns into an opportunistic pathogen and functions as a bridge organism to bind *P. gingivalis* through the fusobacterial adhesins RadD, Fap2 and FomA. *NGS* next-generation sequencing

The microorganisms living in the oral cavity have been referred to as the oral microbiota, oral microflora, or oral microbiome [50, 51]. The homeostasis established by oral microbiota is important in maintaining oral health (Fig. 1) [28, 52]. Over 2000 different microbial taxa, including bacteria, fungi, protozoa, archaea and viruses, have been detected in the human oral cavity [53]. Among them, bacteria are the most abundant taxonomic group and are usually highly organized in oral biofilms on the oral surfaces [54]. Compared to the oral mucosa, the oral biofilms on the dental surface (known as dental plaque biofilms) contain a higher microbial load and are more essential to maintaining oral health, as the oral mucosa are covered by epithelial cells and are frequently shed [54, 55]. By using 16 s ribosomal RNA (rRNA) sequencing, the Human Microbiome Project (HMP) has explored the microbiome in each oral habitat in healthy individuals and found that the predominant genera of the subgingival plaque, in terms of dental plaque biofilm, are *Streptococcus* and *Fusobacterium* whereas the predominant genera of the supragingival plaque are *Streptococcus* and *Capnocytophaga* [56]. With the further extensive application of next-generation sequencing (NGS) techniques, the composition of microbiota related to periodontal health and periodontal disease has also been identified (Fig. 1). For example, the genera *Streptococcus*, *Actinomyces*, and *Granulicatella* are associated with periodontal health, whereas the genera *Synergistes*, *Prevotella* and *Fusobacterium* are associated with periodontitis [57–60]. The NGS-based studies have also revealed that uncultivable and/or rare oral microbiota, such as *Methanobrevibacter oralis*, *Methanobacterium curvum/congolense*, and *Methanosarcina mazeii* are present in both periodontitis and periodontally healthy subjects [61, 62]. Among these attached microbes in dental plaque, *Fusobacterium nucleatum* (*F. nucleatum*) can function as a bridging organism to connect the primary colonizers such as *Streptococcus* and secondary colonizers like *Porphyromonas gingivalis (P. gingivalis*) through the fusobacterial adhesins RadD, Fap2 and FomA [63]. Thus, *F. nucleatum* is a mutualist in healthy periodontal tissue playing a structurally supportive role in dental plaque biofilms but turns into an opportunistic pathogen leading to periodontitis under dysbiotic conditions (Fig. 1).

Studies have identified several species of oral bacteria, mainly gram-negative anaerobes, which are responsible for periodontitis (Fig. 1). These oral bacteria can be classified into five major complexes, namely the green, purple, yellow, orange, and red complexes [64–66]. The “red complex” and "orange complex” are commonly associated with periodontitis. The “red complex” species, consisting of *P. gingivalis*, *Treponema denticola*, and *Tannerella forsythia*, exhibit very strong associations with periodontitis, indicating that these pathogenic bacteria might be the primary pathogens in periodontitis. Many studies have been conducted to investigate the pathogenic mechanisms and characterize the virulence factors of the three pathogens. A “keystone-pathogen hypothesis” has been proposed in which *P. gingivalis* in the periodontium drives the progression of periodontitis [67]. *P. gingivalis*-derived gingipains, phosphoserine phosphatase SerB and lipopolysaccharide (LPS) are potent virulence factors, which can impair the innate host defense systems and induce a destructive response [68]. Gingipains are arginine-specific cysteine proteinases that can cleave complement component C5 into active fragments C5a locally and activate C5a receptors (C5aR) on leukocytes; C5aR signaling then triggers inflammation and subverts leukocyte killing capacity [69]. Phosphoserine phosphatase SerB secreted by *P. gingivalis* contributes to the inhibition of IL-8, thus delaying the recruitment of neutrophils and leading to overgrowth of oral bacterial species [70]. LPS of *P. gingivalis* exhibits specific lipid A structures, which acts as a Toll-like receptor 4 (TLR4) antagonist to inhibit the activation of TLR4-dependent antimicrobial pathways in leukocytes [71]. The subversion of host defense caused by these virulence factors collectively lead to dysbiotic microbiota, inflammation and bone resorption, resulting in a self-feeding ‘vicious cycle’ [67]. Although the “red complex” species exert keystone effects in disruption of periodontal tissue homeostasis, the putative oral bacteria in the “orange complex” that consists of *F. nucleatum*, *Prevotella intermedia*, *Prevotella nigrescens*, and *Peptostreptococcus micros*, have moderately pathogenic effects on the development of periodontitis. For example, *F. nucleatum* can influence the secretion of inflammatory factors, including TNF-α, IL-6 and IL-8, from immune cells or the oral epithelium [72, 73], and therefore modulates the host inflammatory response. *F. nucleatum*can also interacts directly with *P. gingivalis* and increases the invasion ability of *P. gingivalis* [74]. Furthermore, the virulence properties of *P. gingivalis* can be enhanced by another “orange complex” organism: *Peptostreptococcus micros* through promoting the activity of *P. gingivalis* gingpains [75]. In addition to the above bacteria in the “red complex” and “orange complex”, *Aggregatibacter actinomycetemcomitans* (*A. actinomycetemcomitans*) has been shown to cause periodontitis as well [76]. The virulence factors of *A. actinomycetemcomitans* such as leukotoxin, cytolethal distending toxin, and LPS, can cause tissue destruction and bone resorption by promoting inflammation and interacting with the immune system (Fig. 1).

## Regulation of hallmarks of cancer by oral microbiota

**Fig. 2:**
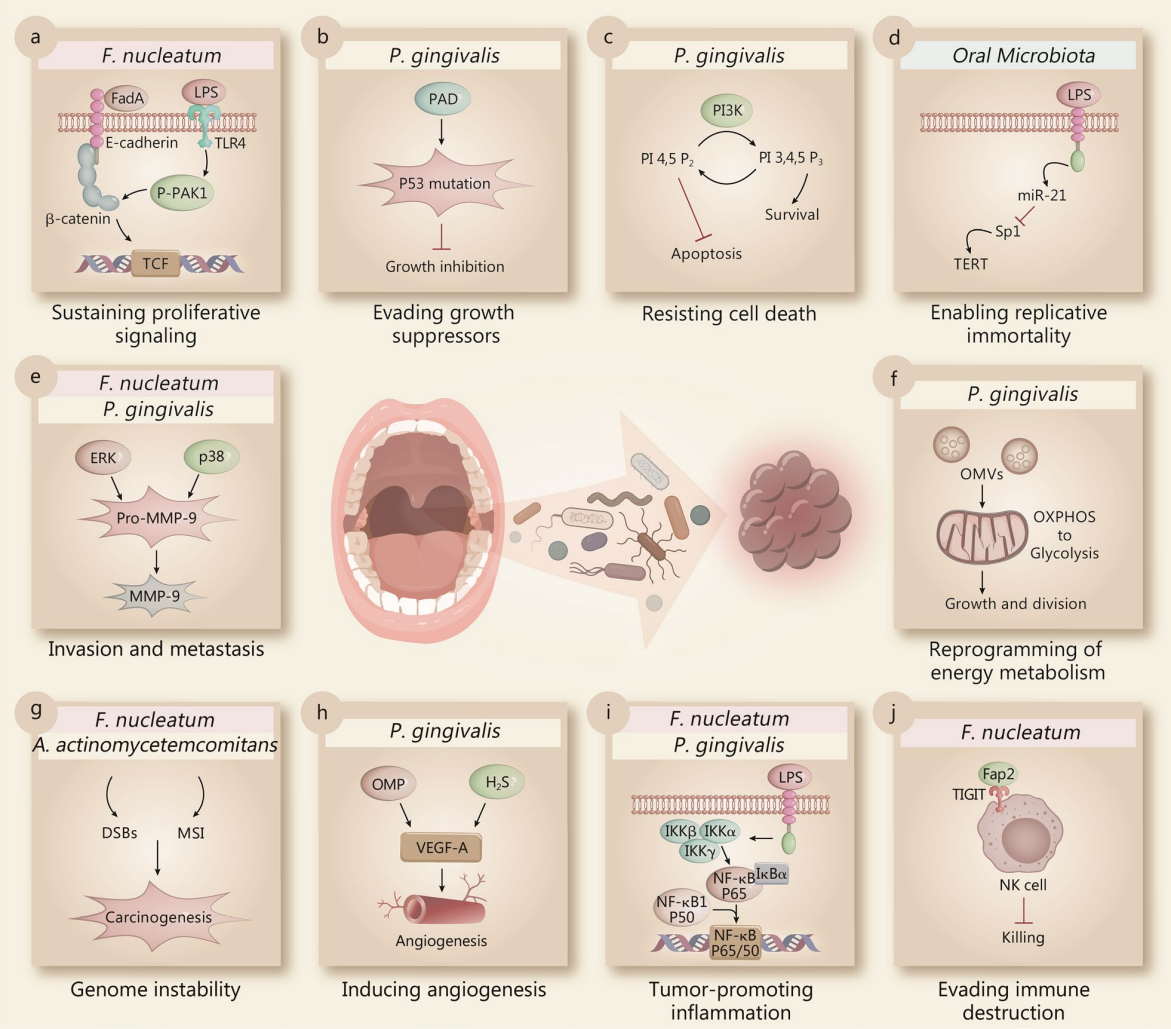
Main effects of individual members of the oral microbiota on hallmarks of cancer. Selected examples of oral microbiota and their molecular mechanisms are shown to be modulating each of the cancer hallmarks, including sustaining proliferative signaling (**a**), evading growth suppressors (**b**), resisting cell death (**c**), enabling replicative immortality (**d**), activating invasion and metastasis (**e**), reprogramming of energy metabolism (**f**), promoting genome instability (**g**), inducing angiogenesis (**h**), inducing tumor-promoting inflammation (**i**), and evading immune destruction (**j**). *DSBs* double-strand breaks, *ERK* extracellular regulated protein kinases, *F. nucleatum Fusobacterium nucleatum*, *H*_*2*_*S* hydrogen sulfide, *LPS* lipopolysaccharide, *miR-21* MicroRNA 21, *MMP-9* matrix metalloproteinase-9, *MSI* microsatellite instability, *OMP* outer membrane protein, *OMVs* outer membrane vesicles, *OXPHOS* oxidative phosphorylation, *P. gingivalis Porphyromonas gingivalis*, *PAD* peptidyl arginine deaminase, *PI3K* phosphatidylinositol 3-kinase, *Sp1* specificity protein 1, *TCF* transcription factor, *TERT* telomerase reverse transcriptase, *TIGIT* T cell immunoglobulin and immunoreceptor tyrosine-based inhibitory motif domain, *TLR4* toll-like receptor 4, *VEGF-A* vascular endothelial growth factor-A

The hallmarks of cancer have been well described by Hanahan and Weinberg, providing us with a remarkable insight into its complex biology [26, 27]. These hallmarks can be classified into two classes: dysfunctional intracellular signaling (including sustaining proliferative signaling, evading growth suppressors, resisting cell death, enabling replicative immortality, activating invasion and metastasis, reprogramming of energy metabolism, and genome instability and mutation) and intercellular signaling creating the tumor microenvironment (including angiogenesis, tumor-promoting inflammation, and evading immune destruction). Many studies have confirmed that oral microbiota could act as potential risk modifiers in the pathogenesis of various malignancies. Indeed, each of the hallmarks of cancer could be modulated by the oral microbiota. *F. nucleatum* and *P. gingivalis* are the two prominent oral bacteria that can modulate the hallmarks of cancer using different mechanisms (Fig. 2).

### Intracellular signaling

Dysregulation of the intracellular signaling pathways of cancer cell leads them to constantly proliferate and evade cell death. Oral microbiota can interact with cells and affect cell proliferation and survival. For example, *F. nucleatum* expresses unique FadA adhesin, which activates β-catenin signaling by binding to E-cadherin [77]. The activation of Wnt/β-catenin signaling promotes colorectal cancer cell proliferation and survival. *F. nucleatum* also activates β-catenin signaling through its LPS via a TLR4/P-PAK1/P-β-catenin S675 cascade in colorectal cancer cells (Fig. 2a) [78].

As well as sustaining proliferative signaling, the ability to evade growth suppression also allows cancer cells to constantly proliferate. The P53 tumor suppressor gene acts as a central node in regulating cell proliferation, which is often down regulated or mutated in cancer cells. *P. gingivalis* may induce mutations of the P53 tumor suppressor via peptidyl arginine deaminase (PAD) enzymes, thus accelerating the progression of the cell cycle in gastrointestinal cancer cells (Fig. 2b) [79]. Apart from *P. gingivalis*, oral bacteria like *Prevotella intermedia*, *Tannerella forsythia*, and *Treponema denticola* also possess the PAD enzyme which induces P53 mutations in pancreatic cancer [80].

In order to be immortal, cancer cells develop the ability to resist cell death. Apoptosis, autophagy and necrosis are three major pathways which control cell death [81]. *P. gingivalis* has been shown to influence cell death decisions by regulating apoptosis (Fig. 2c). Mechanistically, *P. gingivalis* can inhibit apoptosis at the level of mitochondria through the PI3K/Akt pathway [82]. The virulence factors of *P. gingivalis*, such as LPS and fimbriae regulate the PI3K/Akt pathway positively or negatively in different cells [83–86]. The proteolytic activity of gingipains also plays a critical role in disrupting the PI3K/Akt pathway during *P. gingivalis* infection [87]. Another mechanism of *P. gingivalis* in resisting cell death is through manipulation of the JAK/Stat pathway, which induces the intrinsic mitochondrial apoptosis pathway [88]. Accompanied by the changes in cellular signaling pathways, *P. gingivalis* also exerts its antiapoptotic effect by counterbalancing pro- and antiapoptotic members of the Bcl-2 family proteins [89]. The pro-apoptotic molecules such as Bad, Bax, and Bid are down regulated, whereas the anti-apoptotic Bcl-2 is upregulated in the infected cells [90]. The purinergic receptor P2X7 functions as an important mediator of apoptosis and the activation of the P2X7 receptor requires high concentrations of extracellular ATP [91]. *P. gingivalis* can secrete nucleoside diphosphate kinase (NDK), which can scavenge extracellular ATP, thus preventing P2X7-mediated apoptosis [92].

In line with the ability of unlimited proliferation, cancer cells require unlimited replicative potential. The number of cell division cycles in normal cells is limited due to the loss of telomeres after each cell division. In contrast to normal cells, cancer cells have unlimited replicative potential by expressing telomerase, which is able to maintain the stability of telomere DNA. Telomerase Reverse Transcriptase (TERT) is the most important component of the telomerase holoenzyme. Oral microbiota may regulate the expression of TERT via LPS induced miR-21 expression (Fig. 2d) [93].

Activating invasion and metastasis is another complex hallmark capability, contributing to local invasion and distant metastasis. The epithelial-mesenchymal transition (EMT) program broadly promotes invasion and metastasis in cancer cells. Oral bacteria, such as *P. gingivalis* and *F. nucleatum*, have the ability to induce EMT-like features and regulate the expression of markers in vitro [94, 95]. Matrix metalloproteinase-9 (MMP-9) plays an important role in cell migration and invasion by degrading the basement membranes and extracellular matrix. *P. gingivalis* activates ERK1/2-Ets1, p38/HSP27, and PAR2/NF-κB pathways to promote the expression of pro-MMP-9 production. Furthermore, the pro-MMP-9 is subsequently cleaved into its mature active form MMP-9 by gingipains and promotes the invasion of oral squamous cell carcinoma (Fig. 2e) [96]. In addition to *P. gingivalis*, *F. nucleatum* also promotes the secretion of MMP-9, resulting in the activation of cell invasion and metastasis (Fig. 2e) [97].

Reprogramming of energy metabolism is an emerging hallmark of cancer. In order to provide enough energy for cell proliferation, tumor cells need to reprogram energy metabolism, which largely rely on glycolysis. Both *P. gingivalis* and its outer membrane vesicles (OMVs) can shift the energy metabolism from oxidative phosphorylation (OXPHOS) to glycolysis in macrophages (Fig. 2f) [98].

Genomic instability, such as microsatellite instability and chromosomal instability, is an enabling characteristic of most cancers that promotes tumor progression. A cross-sectional study showed that periodontitis could influence the DNA damage status, suggesting that oral microbiota may induce DNA damage and cause genome instability [99]. For example, *A. actinomycetemcomitans* infection can induce DNA double-strand breaks (DSBs) in host cells and subsequently increase the risk of tumorigenesis (Fig. 2g) [100]. *F. nucleatum* is detected in colorectal cancer tissues and is associated with high microsatellite instability (MSI-H) and a high CpG island methylation phenotype (CIMP) (Fig. 2g) [101].

### Intercellular signaling

Angiogenesis is a prominent feature of cancer, which provides nutrients and oxygen for cancer cells and is key to the hematogenous metastatic process. VEGF-A (vascular endothelial growth factor-A) is a potential inducer of angiogenesis. The vesicle and outer membrane proteins (OMP) from *A. actinomycetemcomitans* and *P. gingivalis* are able to stimulate VEGF-A expression levels in human gingival fibroblasts [102]. Oral bacteria (e.g., *P. gingivalis*, *Prevotella intermedia*, *A. actinomycetemcomitans*, *and F. nucleatum*) can produce volatile sulfur compounds (VSCs), such as hydrogen sulfide (H_2_S), to promote angiogenesis that can provide the cancer cells with nutrients and oxygen (Fig. 2h) [103].

Inflammation has long been associated with cancer pathogenesis. Inflammation is stimulated by innate-immune receptors such as the Toll-like receptors (TLRs) [104]. The activation of inflammatory signals creates a tumor environment that promotes cancer formation and growth [105]. The NF-κB signaling pathway plays a vital role in activating a number of inflammatory genes [106]. The LPS from oral bacteria (including *P. gingivalis* and *F. nucleatum*) interacts with TLRs and induces innate immune pathways such as NF-κB signaling pathway (Fig. 2i) [107]. NF-κB signaling then activates several inflammatory bioactive molecules, including IL-6 that sustains proliferative signaling and resists cell death, VEGF that induces angiogenesis, metalloproteases that facilitate invasion and metastasis and reactive oxygen species (ROS) that lead to DNA damage [108]. Recently, Al-Hebshi et al. also suggested a new “passenger-turning-driver” functional model for the role of oral microbes in cancer pathogenesis, in which the oral microbes change their role as passive bystanders ("passengers") to active contributors to the tumorigenesis ("drivers") by sustaining chronic inflammation [109].

Immune surveillance acts as a barrier to tumor formation and progression, however, mounting evidence has shown that cancer cells are able to create an immunosuppressive microenvironment to evade immune destruction [110]. The oral microbiota also take part in tumor-host immunological interactions and facilitate cancer cells to evade immune destruction. For example, natural killer (NK) cell killing of cancer cells can be inhibited by *F. nucleatum*. Fap2 protein of *F. nucleatum* directly interacted with T cell immunoglobulin and immunoreceptor tyrosine-based inhibitory motif domain (TIGIT), which leads to the delivery of an inhibitory signal in NK cells, subsequently leading to the inhibition of NK cell cytotoxicity (Fig. 2j) [111].

## Potential mechanisms of oral microbiota in genitourinary cancers

**Fig. 3:**
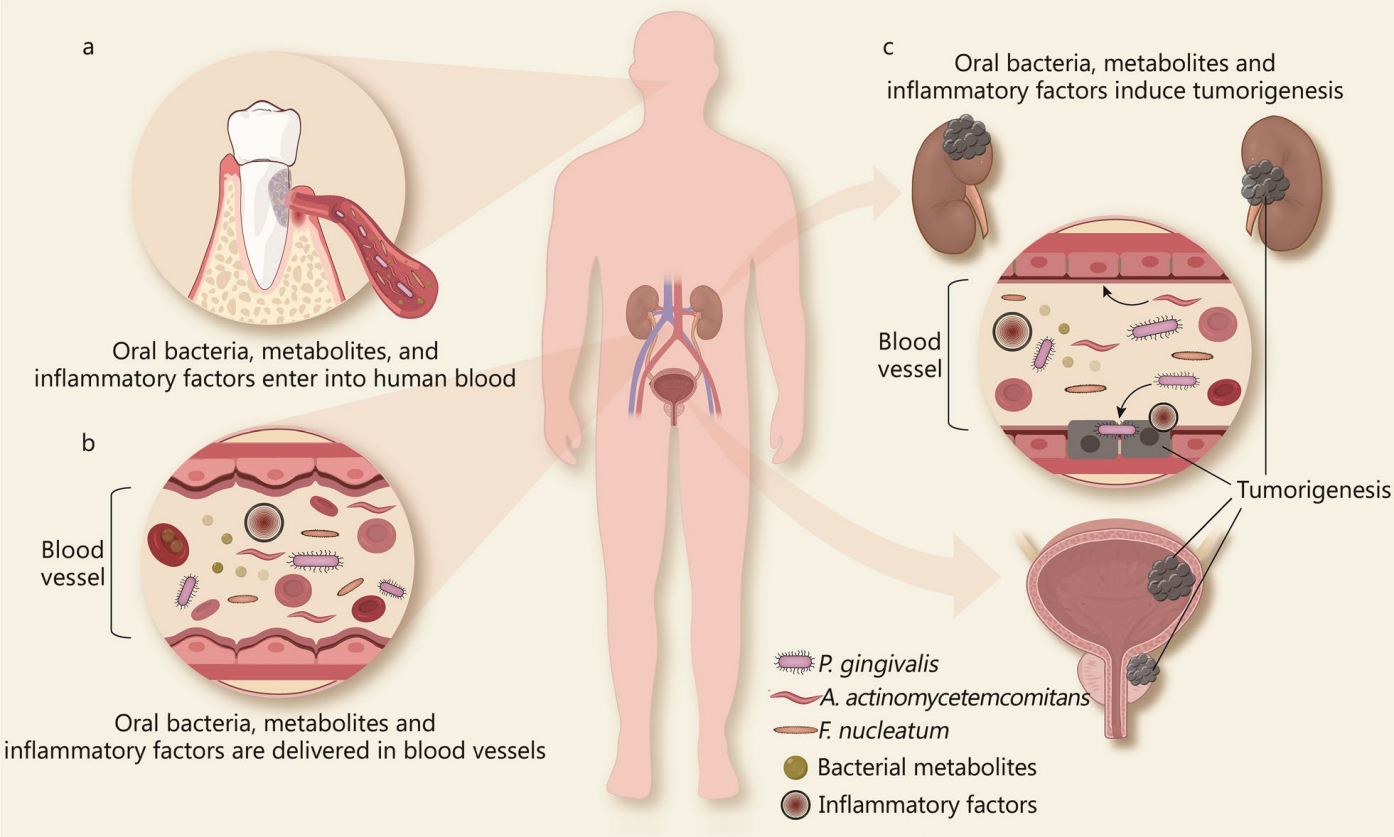
Potential molecular mechanisms by which oral microbiota promote genitourinary cancers. **a** Oral bacteria, bacterial metabolites, and inflammatory factors enter into human blood. **b** Oral bacteria, bacterial metabolites, and inflammatory are delivered via blood vessels. **c** Oral bacteria may colonize the urinary system through blood vessel. Subsequently, the structural components of bacteria (e.g., LPS, FadA) and bacterial metabolites (e.g., gingipains) promote the development of bladder, prostate and kidney cancer. Meanwhile, the long-term presence of inflammatory cells and inflammatory molecules in the systemic circulation is associated with the early formation of primary epithelial tumors in bladder, kidney, and prostate cancers, and promote the excessive proliferation, invasiveness, and angiogenesis of cancer cells. *A. actinomycetemcomitans Aggregatibacter actinomycetemcomitans, F. nucleatum Fusobacterium nucleatum*, *P. gingivalis Porphyromonas gingivalis*

Although oral microbiota has been demonstrated to modulate almost all the hallmarks of cancer in different types of cancers (Fig. 2), the contribution of oral microbiota to genitourinary cancers remains rather mechanistically speculative because genitourinary organs are far away from the periodontium where the oral microbiota originally reside. However, the epidemiological associations of periodontitis with the risk of genitourinary cancers and the detection of oral microbiota in genitourinary organs indicate the presence of the oral-genitourinary axis and oral microbiota may play roles in the initiation and progression of genitourinary cancers. Despite many pieces of evidence exploring the capability of oral microbiota to regulate the hallmarks of cancer, few direct in vitro and in vivo studies have explored the possibility that oral microbiota could influence the development of genitourinary cancers. Therefore, we proposed several causative factors derived from oral microbiota that may act on genitourinary cancers in a direct way through oral bacteria’s structural components or their metabolites and/or an indirect way through systemic inflammation (Fig. 3).

### Direct effect of oral microbiota and its metabolites

The conventional view assumes that the urinary system is sterile. However, recent studies have revealed that microbial populations are present in the urinary system, which are described as the "urinary microbiome". A specific urinary microbiome of urine has been reported among healthy individuals and its composition can change with the progression of genitourinary malignancies [25]. Accumulating evidence supports the spread of the oral bacteria through transient bacteremia, resulting in bacterial colonization in a distant organ. For example, *P. gingivalis*, *A. actinomycetemcomitans* and *Treponema denticola* were detected in atherosclerotic plaques, suggesting that oral bacteria could enter into the bloodstream and colonize distant areas of the cardiovascular system [112]. Placental colonization with oral bacteria, such as *P. gingivalis* and *F. nucleatum,* provides evidence that oral bacteria can cross the placental barrier and cause adverse pregnancy outcomes [113, 114]. Oral *P. gingivalis* infection in mice can cross the blood–brain barrier, leading to brain colonization and the increase of amyloid plaques in the brain, which also supports the association of Alzheimer's disease with chronic periodontitis [115]. In the urinary system, Estemalik et al. found that oral bacteria, such as *P. gingivalis*, *Prevotella intermedia*, and *Treponema denticola*, can be detected in the prostatic secretions of patients with periodontitis [29], suggesting that they may colonize the urinary system via the blood vessels. Subsequently, the structural components of bacteria (e.g., LPS, FadA) and bacterial metabolites (e.g. gingipains) may be involved in promoting the development of prostate, bladder and kidney cancer (Fig. 3). An animal model study has shown that injecting LPS derived from *P. gingivalis* intraperitoneally into mice can induce the overexpression of Saa3, Ticam2, Reg3b, Oxct2a, and Xcr1 in the kidney and these genes regulate a variety of important biological processes, including cell adhesion, apoptosis, immune responses and inflammation, which may regulate the different hallmarks of genitourinary cancers [116]. Several in vitro studies have also shown that *P. gingivalis* and *Treponema denticola* can degrade the epithelial cell–cell junction complexes in Madin-Darby canine kidney (MDCK) cells by regulating EMT markers (like E-cadherin and ZO-1), and therefore may contribute to cell invasion and metastasis of genitourinary cancers [117, 118]. It should be pointed out that even though oral bacteria are found in the urinary system, they may have been passive bystanders brought to the urinary system via bacteremia. More studies are needed to detect the metabolites of oral microbiota in the urinary system in order to claim they have been alive and possibly played a role in genitourinary cancers.

### Indirect effect of systemic inflammation

Periodontitis is known as an inflammatory disease that can lead to systemic inflammation [119]. Chronic inflammation is a well-known risk factor for cancer, which affects its development at different stages [120]. In genitourinary cancers, inflammatory cells (like mast cells, granulocytes, NK cells, and lymphocytes) and inflammatory molecules (like IL-6, IL-1β, TNF-α, and TGF-β) play important roles in the transformation, proliferation, invasion, metastasis, and angiogenesis of cancer cells [121–123]. Of particular note, the progression model for prostate cancer has been proposed, which highlights the vital function of inflammation in prostate cancer [124]. In this model, chronic prostatitis is accompanied with inflammatory cell infiltration, which can lead to proliferative inflammatory atrophy (PIA). PIA may progress to prostatic intraepithelial neoplasia (PIN) under the continuous stimulation of inflammation, and eventually it evolves into prostate cancer. The virulence factors of oral microbiota, such as LPS or gingipains in the oral cavity, could activate innate immune pathways by binding TLR and then induce the activation of a wide range of inflammatory pathways (like NF-κB signaling pathway and PI3K/Akt signaling pathway) in the immune cells. Subsequently, pro-inflammatory molecules such as IL-6, IL-1β, TNF-α, PGE2, or MMPs are secreted into the systemic circulation by immune cells [125]. Meanwhile, inflammatory cells may also enter into bloodstream leading to an inflammatory cell infiltration in a distant organ [126]. The long-term presence of inflammatory cells and inflammatory molecules in the systemic circulation is associated with the early formation of primary epithelial tumors in bladder, kidney, and prostate cancers, and promotes the excessive proliferation, invasiveness, and angiogenesis of cancer cells. Thus, the systemic inflammation processes caused by oral microbiota may partly interpret the crosstalk between periodontitis and genitourinary cancers (Fig. 3).

## Conclusions

The research on the relationship between periodontitis and genitourinary cancers is still in its infancy, with only a handful epidemiological studies available supporting this view. Oral microbiota may regulate the hallmarks of cancer through direct effects (structural components and metabolites) or indirect effects (systemic inflammation), and subsequently promote the tumorigenesis and progression of prostate, bladder and kidney cancer. Culture- and molecular-based detections of oral microbiota in genitourinary cancers shed new light on the direct relationship between periodontitis and genitourinary cancers and may suggest the detection of oral microbiota as new biomarkers to predict genitourinary cancers in patients with periodontitis. Further studies to explore the biological function and underlying mechanisms of oral microbiota in genitourinary cancers would also open new avenues for cancer therapy by targeting oral microbiota.

## Data Availability

Not applicable.

## Abbreviations

*A. actinomycetemcomitans*: *Aggregatibacter actinomycetemcomitans*
Akt: Protein kinase B
ATP: Adenosine triphosphate
Bad: BCL2 associated agonist of cell death
Bax: BCL2-associated X
Bcl-2: B-cell lymphoma-2
Bid: BH3 interacting domain death agonist
C5aR: C5a receptor
CIMP: CpG island methylation phenotype
DSBs: Double-strand breaks
EMT: Epithelial-mesenchymal transition
*F. nucleatum*: *Fusobacterium nucleatum*
H_2_S: Hydrogen sulfide
HMP: Human Microbiome Project
IL-6: Interleukin-6
IL-8: Interleukin-8
JAK: Janus kinase
LPS: Lipopolysaccharide
MDCK: Madin-Darby canine kidney
MMP-9: Matrix metalloproteinase-9
MSI-H: High microsatellite instability
NDK: Nucleoside diphosphate kinase
NGS: Next-generation sequencing
NK: Natural killer
NCI: National Cancer Institute
OMP: Outer membrane protein
OMVs: Outer membrane vesicles
OXPHOS: Oxidative phosphorylation
*P. gingivalis*: *Porphyromonas gingivalis*
P2X7: P2X Purinoceptor 7
PAD: Peptidyl arginine deaminase
PI3K: Phosphatidylinositol 3-kinase
PIA: Proliferative inflammatory atrophy
PIN: Prostatic intraepithelial neoplasia
ROS: Reactive oxygen species
rRNA: Ribosomal RNA
Stat: Signal transducer and activator of transcription
TERT: Telomerase Reverse Transcriptase
TIGIT: T cell immunoglobulin and immunoreceptor tyrosine-based inhibitory motif domain
TLR4: Toll-like receptor 4
TLRs: Toll-like receptors
TNF-α: Tumor necrosis factor alpha
VEGF-A: Vascular endothelial growth factor-A
VSCs: Volatile sulfur compounds
